# Anticoagulation Management for Veno-Venous ECMO in COVID-19 Patients: Argatroban as Rescue Therapy in Heparin-Associated Thrombocytopenia

**DOI:** 10.3390/jcm13226984

**Published:** 2024-11-20

**Authors:** Lorenzo Schiavoni, Alessia Mattei, Martina Cuccarelli, Alessandro Strumia, Carmelo Dominici, Antonio Nenna, Jessica Aceto, Gloria Palazzo, Giuseppe Pascarella, Fabio Costa, Rita Cataldo, Felice Eugenio Agrò, Massimiliano Carassiti

**Affiliations:** 1 Unit of Anesthesia and Intensive Care, Fondazione Policlinico Universitario Campus Bio-Medico, 00128 Rome, Italy; l.schiavoni@policlinicocampus.it (L.S.); a.mattei@policlinicocampus.it (A.M.); martina.cuccarelli@unicampus.it (M.C.); a.strumia@policlinicocampus.it (A.S.); jessica.aceto@unicampus.it (J.A.); g.pascarella@policlinicocampus.it (G.P.); f.costa@policlinicocampus.it (F.C.); r.cataldo@policlinicocampus.it (R.C.); m.carassiti@policlinicocampus.it (M.C.); 2 Unit of Cardiac Surgery, Fondazione Policlinico Universitario Campus Bio-Medico, 00128 Rome, Italy; c.dominici@policlinicocampus.it (C.D.); a.nenna@policlinicocampus.it (A.N.); 3 Operative Research Unit of Transfusion Medicine and Cellular Therapy, Fondazione Policlinico Universitario Campus Bio-Medico, 00128 Rome, Italy; g.palazzo@policlinicocampus.it

**Keywords:** SARS-CoV-2, ECMO, heparin, HIT, argatroban, COVID-19, extracorporeal membrane oxygenation, thrombocytopenia

## Abstract

**Background/Objectives:** Extracorporeal membrane oxygenation (ECMO) has been widely used as a life support technique in COVID-19 acute respiratory distress syndrome (ARDS). The use of anticoagulation during ECMO support remains a topic of debate. The primary aim of this study is to demonstrate the safety and efficacy of using argatroban as an anticoagulant instead of heparin in patients with heparin-associated thrombocytopenia. **Methods:** 40 patients were enrolled and initially treated with unfractionated heparin for anticoagulation during ECMO, composing the UFH group. Twenty-one of these patients experienced a drop in platelet count to below 100,000 cells/mm^3^ and, after testing negative for IgG anti-PF4/heparin, the anticoagulation was switched to argatroban, composing the ARG group. Hemorrhagic events were recorded along with blood chemistry parameters. **Results:** Bleedings were significantly more frequent in the UFH group than in ARG group (58/579 days vs. 21/357 days, *p* = 0.041). No significant differences were observed in hemorrhagic episodes for each bleeding site, except for tracheal stoma (14 vs. 1, *p* = 0.011). No differences in activated partial thromboplastin time (aPTT) values were found between the two groups (aPTT 42.65 s vs. 44.70 s, *p* = 0.443). Linear regression analysis revealed that the platelet count on day 5 was correlated with the initial platelet count but not with the type of anticoagulant used (*p* = 0.001, CI 0.55, 0.69 and *p* = 0.078). Linear regression analysis in both groups showed a correlation between the duration of ECMO support and intensive care unit stay for the median aPTT and median platelet count. Furthermore, no major systemic thrombotic events or circuit clotting were observed in this patient cohort. **Conclusions:** Argatroban seems to be safe in patients with persistent heparin-associated thrombocytopenia undergoing ECMO.

## 1. Introduction

Among patients suffering from pneumonia related to SARS-CoV-2 infection, approximately 20% develop a severe condition of ARDS (acute respiratory distress syndrome) that requires admission to the ICU (intensive care unit) to initiate invasive support. In the most life-threatening conditions, ECMO (extracorporeal membrane oxygenation) support is employed as a rescue therapy and an adjunct strategy to mitigate lung injury resulting from high-pressure mechanical ventilation [1,2].

The international standard for anticoagulation is unfractionated heparin due to its cost-effectiveness, widespread availability, titratability, and the potential reversibility of its effects [3,4].

Thrombocytopenia is a common adverse event occurring during heparin or heparin-like drug infusion. Based on the underlying pathophysiological mechanism, it is divided into two different types: type 1 and type 2 heparin-induced thrombocytopenia (HIT). Type 1 HIT (also known as heparin-associated thrombocytopenia) is a dose-related response of platelets to heparin infusion and is the consequence of platelet aggregation promoted by heparin, with clamping and clearance in the spleen and liver. Type 2 HIT is a rare, immune-mediate, transient prothrombotic condition. In selected patients, heparin exposure induces the formation of IgG-PF4-heparin complexes, which can promote platelet activation and aggregation [5].

New options for alternative anticoagulation, such as direct thrombin inhibitors (argatroban, bivalirudin, lepirudin, and desirudin), fondaparinux, and danaparoid [6], have been identified to overcome this threatening hurdle.

The latest ELSO guidelines reported the use of direct thrombin inhibitors (DTI) as an off-label management for anticoagulation, even if the ELSO expert panel considered them an efficient alternative to unfractionated heparin for adult and pediatric patients [7].

Argatroban maintains its main indication in patients with a moderate to high risk of type 2 HIT, but it is still used off-label for anticoagulation in ECMO. It is usually dosed, in accordance with its drug datasheet, at 2 µg/kg/min; however, guidelines recommend closely monitoring the aPTT, the activated clotting time, with anti-Xa assays, or through viscoelastic point-of-care tests.

In order to understand the potential benefits of argatroban versus UFH, we have decided to compare patients at high hemorrhagic risk for severe thrombocytopenia managed with argatroban to those with a normal platelet count who are managed with unfractionated heparin.

The primary aim of our study is to compare the incidence of hemorrhagic events under anticoagulant treatment in patients treated with argatroban versus unfractionated heparin.

The secondary aims of this study are to evaluate the protective effect of argatroban versus unfractionated heparin on platelet count and its correlation with days on ECMO and ICU length of stay.

## 2. Materials and Methods

### 2.1. Study Design

This is a retrospective, monocentric study conducted at the Fondazione Policlinico Universitario Campus Bio-Medico in Rome.

This study was designed following the STROBE guidelines for observational cohort studies.

**Figure 1:**
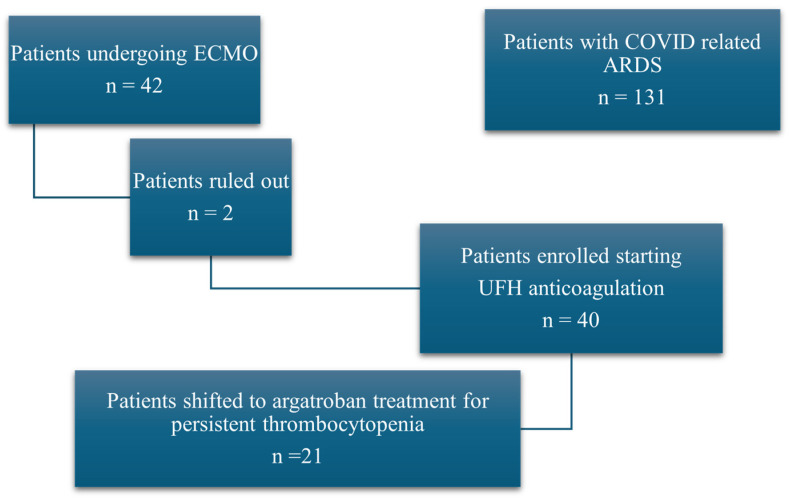
STROBE flow chart for patient selection and subgrouping. Abr.: acute respiratory distress syndrome (ARDS), extracorporeal membrane oxygenation (ECMO), coronavirus disease (COVID), unfractionated heparin (UHF).

We included patients hospitalized between the 1st of November 2020 and the 11th of July 2021 who were admitted to the ICU because of COVID-19-related bilateral interstitial pneumonia (Figure 1).

Inclusion criteria were:

- Patient age ≥ 18 years;
- Acute hypoxic respiratory failure;
- VV-ECMO support.

Exclusion criteria were:

- VV-ECMO run lasting for less than 5 days;
- Any absolute contraindication to anticoagulation, as reported by the ELSO guidelines.

All patients enrolled in the study entered into the UFH group (treated with unfractionated heparin from the beginning of the ECMO run). In case of a platelet count drop to values below 100,000 cells/mm^3^, heparin was suspended and replaced with argatroban, thus making the involved patient(s) leave the UFH group to enter the ARG group (treated with argatroban until weaning from ECMO or until death).

All the patients involved were intubated and under sedation and invasive mechanical ventilation.

### 2.2. ECMO Support

We applied different kinds of VV-ECMO circuits, such as: Ecmolife Eurosets in 6 patients, MAQUET MEDOS Deltastream-HC in 10 patients, free life FT2800 pro in 3 patients, and MAQUET HU 35 in 21 patients. Each ECMO ran with a femoral–jugular configuration, with a drainage cannula in the femoral vein and a reinfusion cannula in the right internal jugular vein. We started the anticoagulation therapies with unfractionated heparin while maintaining an aPTT 1.5–2 times above the normal value (50–70 s instead of the normal range), measured every 6 h; antithrombin levels were measured once daily.

If platelet count fell to <100,000 cell/mm^3^, we switched to an anticoagulation therapy using argatroban (aPTT maintained at 1.5 to 2 times above normal values), measured every 6 h, and patients were then screened for heparin-PF4 IgG with an automatic quantitative test for detection of anti-heparin/PF4 antibodies that was provided by ACL AcuStar^TM^ (Wefern, Instrumental Laboratory Spa, Milan, Italy).

### 2.3. Study Outcomes

The primary aim of this study was to assess the reduction in overall risk for bleeding events in patients who received anticoagulation treatments with argatroban versus unfractionated heparin.

When moderate thrombocytopenia rose, all patients were screened for HIT with an automatic quantitative test for detection of anti-heparin/PF4 antibodies, provided by ACL AcuStar^TM^.

This aim was pursued by comparing the frequencies of major hemorrhagic complications, classified according to recommendations by ELSO as a drop of hemoglobin >2 g/dL/day, transfusion of >2 packed red cells in 24 h, or retroperitoneal, cerebral, or pulmonary bleeding. Minor bleedings were classified as <2 packed red cells in 24 h.

Bleeding events were classified into intracranial, pulmonary, gastroenteric, or intermuscular bleeding or bleeding occurring in the high respiratory system, the venous cannulation areas, or around the tracheal stoma. We decided to collect data on every bleeding that was unresponsive to conventional local management and that lasted for more than 12 h with a potential risk to patients. These data were collected independently from hemodynamic instability or the need for blood transfusion.

The secondary measured outcome was the influence of anticoagulation management on platelet count.

In order to compare data, platelet count, PT, aPTT, and D-dimers were collected as medians for the whole stay.

To evaluate the impact of anticoagulant infusion, we defined a platelet trend calculated on the difference between platelet count at day 5 (PLT T5) and at day 0 (PLT T0) of the anticoagulant infusion, and matched differences were recorded. All records were screened for thrombotic events.

Platelet transfusion was administered, following recent guidelines, when a severe bleeding with hemodynamic instability occurred or in case of a platelet count inferior to 50,000 cell/mm^3^.

Tranexamic acid was used for the management of uncontrolled bleeding despite platelet transfusion and suspended anticoagulant infusion, in accordance with the recommendations made by the ELSO guidelines.

### 2.4. Data Collection

Data were collected using the Ascom Digistat Suite (version 8.2.2.0) and Lutech W-Hospital (version 2.0). Each patient was assigned a progressive number, and data were transferred and stored in an anonymized database using Microsoft Office Excel software.

We collected data on ECMO patients’ age, sex, body mass index (BMI), comorbidities, blood products transfused, ICU length of stay (LOS), days under ECMO and anticoagulation therapy, and weaning from ECMO or death.

### 2.5. Statistical Analysis

Statistical analysis was conducted using Stata Software version 17, and a *p*-value < 0.05 was considered to be statistically significant. Categorical variables are expressed as frequencies and percentages and are compared using the chi-squared test or Fisher’s exact test, as appropriate. Continuous variables were checked for normality with the Kolmogorov–Smirnov test. Normally distributed variables are shown as means and standard deviations and compared with parametric tests (Student’s *t*-test). Non-normally distributed variables are presented as medians and interquartile ranges and compared with non-parametric tests (Mann–Whitney U test). Regression analysis was performed for continuous outcomes using linear regression to estimate the effect of the baseline platelet count/coagulation test and the group effect (*regress* command in STATA). R-squared and residuals analysis (*rvfplot* command in STATA) were used to assess model fit. Statistical analysis was performed with STATA version 17. A two-tailed *p*-value < 0.05 was considered statistically significant.

### 2.6. Ethical Approval

This retrospective observational study did not require any patient consent and was approved by the Ethical Committee of the Fondazione Policlinico Universitario Campus Bio-Medico with the number OSS/22.46.

Given the retrospective design of the study, according to Italian national regulations, the Ethical Committee approved the waiver of informed consent.

## 3. Results

While collecting data, 131 patients were admitted to our ICU department due to ARDS resulting from COVID-19 pneumonia. A total of 91 patients were excluded: 2 patients died within 5 h after the initiation of ECMO, and 89 patients did not require extracorporeal membrane oxygenation. Ultimately, the study enrolled 40 patients, consisting of 31 men (77.5%) and 9 women (22.5%).

We successfully weaned 16 patients completely from VV-ECMO, while 24 patients died due to worsening ARDS. All 40 patients were included in the UFH group (treated with unfractionated heparin). Among them, 21 patients (52.5%) started a continuous infusion of UFH but were later switched to argatroban due to a significant drop in platelet count associated with the UFH therapy, with a median drop of −127,762 (98,182) cells/mm^3^. The remaining 19 patients completed their ECMO course under continuous infusion of unfractionated heparin.

Population characteristics are summarized in Table 1. A statistically significant difference was observed in the duration of ECMO therapy, with the UFH group averaging 10 days (IQR 8–26) and the ARG group averaging 31 days (IQR 22–47), yielding a *p*-value of 0.0008. Similarly, the length of ICU stays differed significantly, with the UFH group averaging 31 days (IQR 18–39) and the ARG group averaging 43 days (IQR 32–56), resulting in a *p*-value of 0.028. No differences were found in the duration of anticoagulation therapy. Overall mortality rates did not differ significantly, despite twice as many patients dying in the argatroban group (16 in the ARG group vs. 8 in the UFH group).

### 3.1. Bleeding Events

The frequency of bleeding events is reported in Table 2, detailing both absolute counts and percentages of affected patients. In the UFH group, a total of 58 bleeding episodes were recorded, whereas the ARG group had 21 episodes. Considering the differences in group sizes, the absolute frequency of bleeding events was normalized to the total days of ECMO for each group. This analysis revealed that patients under UFH had a bleeding risk of approximately 0.14 per day on ECMO, compared to a risk of 0.03 per day for patients under argatroban (*p* = 0.041). Chi-square analysis confirmed this result was statistically significant.

Sub-analysis of bleeding sites indicated no statistical differences in bleeding episodes, except for tracheal stoma bleeding, which had 14 events in the UFH group versus 1 in the ARG group (OR 7.29; *p* = 0.011).

### 3.2. Coagulation Monitoring

As a secondary aim, we studied platelet counts and changes in coagulation parameters (aPTT, PLT count, and D-dimers) collected through blood tests. We quantified the initial drop in platelet counts during the first five days of therapy with heparin and argatroban. The median aPTT was similar between groups, with a median of 42.65 (IQR 37.25; 46.63) seconds in the UFH group and 44.70 (IQR 36.70; 48.20) seconds in the ARG group. However, the median platelet count differed significantly, with a median of 138,500 (IQR 100,000; 185,250) cells/mm^3^ in the UFH group and 80,000 (IQR 52,500; 107,500) cells/mm^3^ in the ARG group (*p* < 0.001).

At day 0, the median platelet count was significantly different between groups, measuring 271,000 (IQR 188,500; 350,000) cells/mm^3^ in the UFH group and 59,000 (IQR 42,000; 85,000) cells/mm^3^ in the ARG group (*p* < 0.001). By day 5, the median platelet count was also significantly different, with 177,500 (IQR 115,000; 250,500) cells/mm^3^ in the UFH group and 67,000 (IQR 46,000; 114,000) cells/mm^3^ in the ARG group (*p* < 0.001). The median platelet count drop over the first five days of treatment was significantly different, with a median drop of −72,500 (IQR −135,500; −48,000) cells/mm^3^ in the UFH group and 7000 (IQR −3000; 16,000) cells/mm^3^ in the ARG group (*p* < 0.001). All results are detailed in Table 3.

None of the patients tested positive for heparin-PF4 IgG, and none experienced major thrombotic events. Heparin use was associated with a statistically significant mean drop in platelet count during the first five days compared to argatroban; however, patients who switched anticoagulants exhibited moderate thrombocytopenia. A sub-analysis of platelet count drops during heparin therapy compared patients who continued heparin with those who switched to argatroban, revealing a non-significant difference in platelet counts (−127,762 cells/mm^3^ vs. −69,526 cells/mm^3^).

### 3.3. Blood Products Transfused

The two groups differed significantly in terms of the total amount of platelet units and fresh frozen plasma transfused. The total amount was weighed on the day of infusion of each anticoagulant.

In particular, a total of 6 platelet units were transfused over 579 days of heparin infusion versus 18 platelet units over a period of 357 days in the ARG group (OR: 4.9, *p* < 0.001).

Similarly, transfused units of fresh frozen plasma were significantly lower in the UFH group than in ARG group (16/579 unit/days versus 18/357 unit/days, OR: 1.82, *p* = 0.082).

On the other hand, patients in both groups presented the same risk to be transfused with red blood cell units (254 versus 167, OR: 1.06, *p* = 0.593).

### 3.4. Linear Regression Analysis

A regression analysis was conducted on PLT T5 to assess its correlation with either anticoagulant used or PLT T0. The analysis showed that the statistically significant drop in PLT was correlated only with the initial platelet count and was independent of the type of anticoagulant (Table 4).

**Figure 2:**
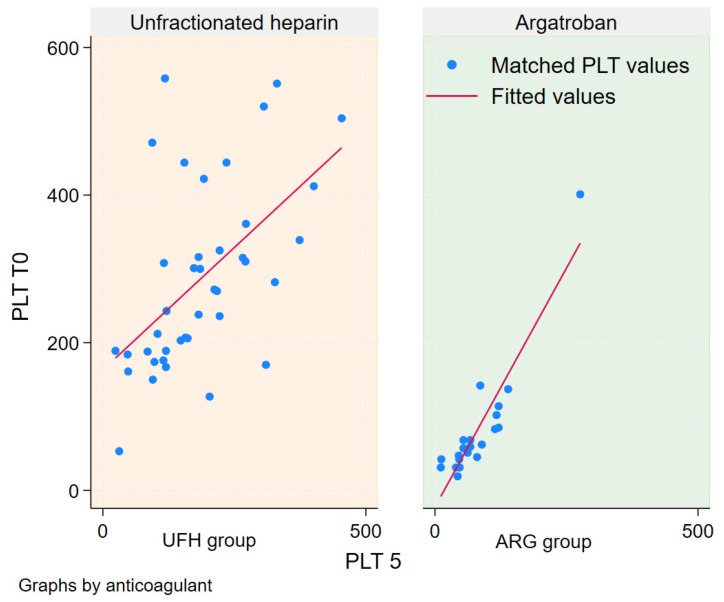
Linear regression of PLT T5 on PLT T0 for subgroups. Blue dots are correlation points of PLT 5 to PLT 0, the red line represents the linear regression for both groups.

**Figure 3:**
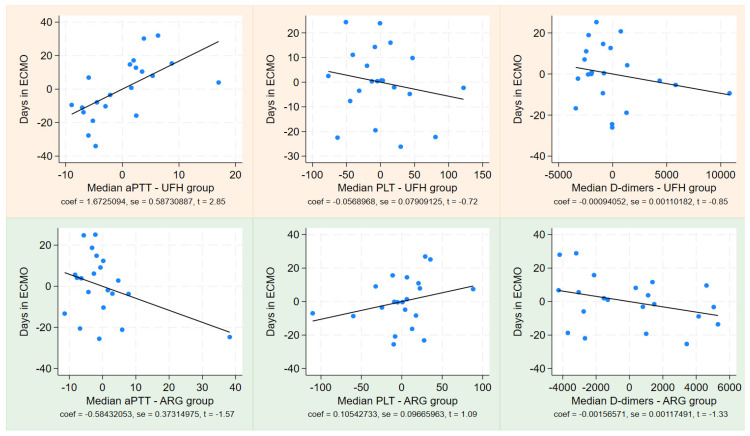
Linear regression between days in ECMO and the aPTT, PLT, and D-dimer in both groups—light orange for the UFH group and light green for the ARG group. ECMO = extracorporeal membrane oxygenation.

**Figure 4:**
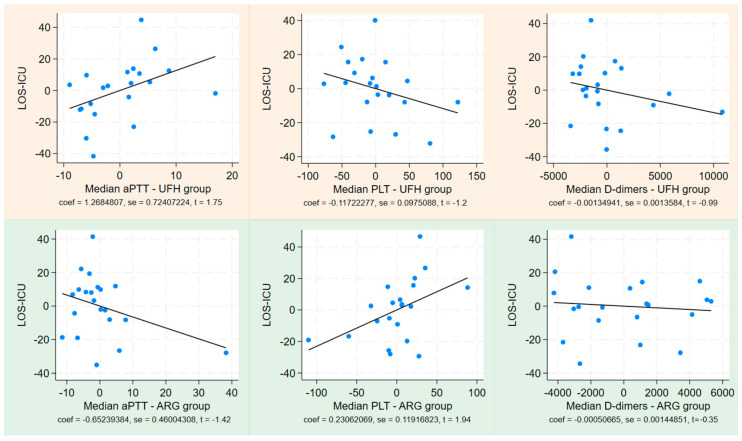
Linear regression between LOS-ICU and PTT, PLT, and D-dimer in both groups—light orange for the UFH group and light green for the ARG group. aPTT = activated prothrombin time, PLT = platelet, UFH = unfractionated heparin, ARG = argatroban, LOS-ICU = length of stay in intensive care unit.

Figure 2 illustrates the linear regression of PLT T5 matched to PLT T0 in both subgroups. Separate linear regression analyses were performed using median PLT, median aPTT, and median D-dimer values as independent variables for LOS and ECMO days, as displayed in Figure 3 and Figure 4. This analysis revealed a positive correlation between LOS and ECMO days with the median aPTT in the heparin group, and a negative correlation with median PLT count in the same group. Conversely, in the argatroban group, LOS and ECMO days correlated negatively with the median aPTT and positively with the median PLT count. No significant correlations were found for D-dimer values.

A linear regression analysis of PLT T5 as a dependent variable, with PLT T0 and the anticoagulant used as independent variables, indicated that PLT T5 depended on PLT T0 but not on the type of anticoagulant (*p* = 0.001, CI: 0.55, 0.69 and *p* = 0.078, CI: −3.51, 64.5; Table 4 and Figure 2).

Among the 40 patients, 61 ECMO circuits were used, with a median duration of 24 days (IQR 9–36.25), totaling 1049 days. A total of 21 circuit and oxygenator changes were made for 17 patients, primarily due to oxygenator exhaustion. Notably, there was one case of clotting on the outflow side of the oxygenator and one case of bacterial contamination (confirmed by blood cultures from the oxygenator). Twelve changes occurred under heparin and nine under argatroban; however, a statistical analysis for correlation would be misleading, as several patients had ECMO support suspended before oxygenator exhaustion, and the study was not designed to capture this information.

## 4. Discussion

This is the first study that explores the incidence of bleeding events in patients undergoing veno-venous ECMO and evaluates the possible correlation to platelet count and anticoagulation management, comparing argatroban to unfractionated heparin. Our results indicate a lower rate of bleeding in patients treated with argatroban compared to those receiving heparin infusion. Additionally, we observed a significantly lower frequency of hemorrhage specifically at the tracheal stoma site. The incidence of bleeding in the gastrointestinal tract, intramuscular sites, upper respiratory tract, cannulation sites, intracranial regions, and pulmonary sites was similar in both groups. However, a slightly lower frequency of bleeding events was noted in the upper respiratory tract and at the cannulation sites, although there was no statistically significant difference. Data suggest that argatroban may be associated with an increased risk of parenchymal bleeding (in the brain and lungs), while unfractionated heparin appeared to correlate more with bleeding at invasive procedure sites (cannulation site, tracheal stoma, upper respiratory tract). However, other risk factors may have interfered with this result, preventing clear conclusions.

This finding was noted despite the significant difference in platelet count between groups, suggesting that low platelet count may not be a major risk factor for bleeding during veno-venous ECMO.

Although our sample size was small, our data indicates a generally similar risk of bleeding events between unfractionated heparin and argatroban. Notably, the total number of bleeding events in the UFH group (58 events over 401 days of ECMO) was significantly higher than in the ARG group (21 events over 648 days of ECMO).

As identified by Chen et al., among twenty-three original research papers, only four retrospective trials directly compared argatroban to UFH in veno-venous ECMO, but the incidence of bleeding and thrombosis was only reported by Fisser et al. [6,8,9,10,11].

Fisser and colleagues directly compared bleeding incidence at various sites between anticoagulant treatments and found no statistically significant difference between argatroban and unfractionated heparin [8], suggesting that argatroban could potentially have a lower impact on platelet count during veno-venous ECMO [8]. We must consider the possibility that this observation may be influenced by biases in our study design.

Sattler et al., in their observational study, decided to change the anticoagulation strategy from UFH to argatroban or vice versa based on suspected drug resistance. They found that argatroban and UFH were similar in effectiveness and complication rate, but argatroban was superior in reaching the anticoagulation target aPTT [12].

Furthermore, we investigated the incidence and severity of thrombocytopenia as a secondary outcome and were able to demonstrate a positive association between argatroban infusion and platelet count compared to heparin. However, the platelet count at the time of the drug switch was significantly lower than at the beginning of heparin infusion.

Wilcoxon rank-sum tests revealed statistically significant differences in platelet count, baseline platelet count (T0), platelet count at day 5 (T5), change in platelet count, and D-dimer levels.

Baseline platelet count in patients starting heparin versus those starting argatroban differed due to several factors. First of all, the platelet count in patients receiving argatroban was likely reduced due to previous anticoagulant treatment, around 11 days of extracorporeal membrane oxygenation support, and a longer duration of COVID-19 infection. For these reasons, this difference should be confirmed through other prospective studies.

Similarly, the statistically significant difference in platelet count observed on day 5 of treatment could result from the longer duration of ECMO support and COVID-19 infection. Consequently, we evaluated the drop in platelet count from day 0 to day 5 of infusion, which revealed a statistically significant difference. This finding may suggest a protective effect of argatroban on platelet count compared to heparin, while also confirming that argatroban is not inferior.

In a recent meta-analysis on platelet recovery in patients undergoing systemic anticoagulation with DTI for HIT, argatroban was found to be successful in achieving complete platelet recovery. Moreover, in this meta-analysis, the efficacy and safety outcomes were comparable among various non-heparin anticoagulants [6].

As Martucci et al. suggested in their recent publication of the PROTECMO study, maintaining the activated partial thromboplastin time (aPTT) at the lower end of the recommended range may be more beneficial than switching anticoagulants [13].

The evident low platelet count in the ARG group could be attributed to heparin-associated thrombocytopenia (HAT or Type 1 HIT); however, in our study, the reduced platelet drop in the ARG group may relate to preexisting thrombocytopenia, to platelet transfusion, or to other uninvestigated conditions. This hypothesis is supported by the linear regression analysis of platelet counts at T5 compared to T0 and the type of anticoagulant, which revealed a stronger correlation between T5 platelet counts and initial counts than to the type of anticoagulant used.

On the other hand, our reported blood product consumption revealed a statistically significant difference in the transfusion of platelet units and fresh frozen plasma. In our opinion, this difference is related more to the need to maintain adequate rheologic conditions during ECMO runs, as suggested by ELSO guidelines. In fact, patients in the ARG group were enrolled because of thrombocytopenia worsening, and prophylactic platelet transfusions are recommended by ELSO guidelines to maintain platelet counts over 80,000 cells/mm^3^.

Differently, Fisser demonstrated a reduced consumption of platelet packs per day in patients treated with argatroban compared to unfractionated heparin since the veno-venous ECMO start, suggesting that this drug may help to preserve platelets during veno-venous ECMO [8]. Similarly, in a recent study involving 57 patients, Menninger et al. showed that argatroban was superior to heparin alone in preserving the oxygenator from exhaustion and in reducing the transfusion rate of blood products [10].

In a cost-effectiveness study, Cho et al. suggested that the longer life of the oxygenator during ECMO in patients treated with argatroban compared to UFH could be attributed to the reduced platelet adhesion to circuit surfaces induced by argatroban [9]. However, no definitive agreement has been found and unfractionated heparin remains the first-line drug for anticoagulation management during ECMO, mainly for its cost-effectiveness ratio and availability.

These data suggest that bleeding events in patients undergoing ECMO are not directly related to thrombocytopenia or to the drug used to manage anticoagulation, but many other patient-related or external factors could interfere with rheologic homeostasis.

The difference in platelet drop between the two groups is noteworthy and supported by a linear regression analysis examining the relationship between median platelet counts and both ECMO days and length of stay (LOS).

This analysis revealed a negative correlation between ECMO days/LOS and platelet counts in the UFH group. We believe this evidence may be related to the fact that patients with shorter ECMO durations and hospital stays tended to survive and were more easily weaned. Conversely, those with longer ECMO runs and extended hospitalizations experienced more complications, and the lower median platelet counts may reflect several factors, including sepsis, circuit wear, or oxygenator degeneration.

In contrast, the linear regression analysis in the ARG group indicated a positive correlation between median platelet counts and ECMO days/LOS-ICU. We hypothesize that this phenomenon may result from two different mechanisms: firstly, argatroban does not promote dose-dependent platelet aggregation in the manner that heparin does—by activating splenic clearance; secondly, argatroban has a protective effect on the oxygenator and circuit, as demonstrated in previous studies, even though, in our study, ECMO runs with argatroban were significantly longer than those with UFH [8,10].

In this study, there was no statistically significant difference in mortality between groups, despite 16 deaths in the ARG group compared to 8 in the UFH group. This discrepancy can be explained by the fact that sicker patients with longer ECMO runs were switched from UFH to ARG, consequently increasing the mortality rate in the ARG group, since run time is known to be an independent variable affecting mortality during ECMO support.

Differently, Rivosecchi et al. evidenced a lower mortality rate associated with a lower incidence of extracorporeal thrombotic events in patients managed with bivalirudin [14]. Similar results have been obtained by Pieri et al., but this study did not highlight a statistically significant difference [15].

Recent annual ELSO guidelines report that clots in the oxygenator occur in nearly 13% of patients. Therefore, continuous pharmacologic anticoagulation is recommended to ensure the proper use of extracorporeal membrane oxygenation support by counteracting the effects of exposure to the non-endothelial surfaces of the ECMO circuit, inhibiting the activation of platelets, coagulation, and inflammatory pathways without increasing the risk of bleeding, even if there is emerging evidence that, in the case of severe thrombocytopenia, a veno-venous ECMO could run without anticoagulation without increasing the risk of thrombo-embolic events [16,17,18].

Despite the potential collateral effects associated with the application of ECMO circuits, ELSO guidelines advocate for systemic anticoagulation therapy for all patients undergoing ECMO support. While the guidelines consider unfractionated heparin as the first-choice anticoagulant, they also do not exclude the use of direct thrombin inhibitors (DTIs) as second-line options, particularly in specific situations, such as confirmed type 2 HIT. Among the DTIs, the literature has explored the efficacy of bivalirudin, lepirudin, and argatroban, with ELSO guidelines recognizing these anticoagulants as potentially useful in an off-label context [19]. Notably, argatroban has been highlighted as a drug capable of inducing and maintaining therapeutic anticoagulation in cases of confirmed or suspected type 2 HIT [20,21].

In contrast to the data reported in the literature, our study observed a lower incidence of circuit thrombosis and oxygenator clotting compared to previous reports, even though the median duration of circuit and oxygenator use was longer and the median aPTT ratio was close to 1.5 times the normal ratio [22]. This evidence suggests that anticoagulation in veno-venous ECMO may be effective at lower doses for prophylaxis [10,11,22,23].

Data presented in Table 1 show no significant differences between the characteristics of the groups, except for ECMO days and LOS in the ICU. This difference arose from our clinical protocol for anticoagulant management, which authorizes intensivists to suspend heparin in case of progressive platelet decrease and to start argatroban infusion to protect residual platelet counts. Statistical analyses on this aspect were deemed unnecessary, as they represent a bias; patients in the ARG group necessarily experienced longer ECMO runs and total LOS due to the duration of heparin treatment followed by argatroban therapy.

## 5. Limits of the Study

Our study is not free of limitations.

Its first weakness can be found in the design: it is a retrospective, monocentric study and, as in other existing studies, the treatment strategy was not decided before the study started; results could, consequently, be warped.

Another limitation is a relatively small sample, which is due to difficulties in recruiting we faced during the critical pandemic event.

One more potential frailty lies in our internal clinical protocol for anticoagulant management therapy: all patients enrolled for anticoagulation with argatroban were previously treated with unfractionated heparin.

Moreover, patients who underwent argatroban infusion due to lowering platelet count suffered from a mean length of stay and ECMO treatment of more than 15 days, and direct comparison of drug impact is thus skewed.

Several causes could have induced low platelet count in our patients, and most of them were time-related, such as sepsis, COVID-19 infection, and mechanical destruction induced by ECMO. However, our results suggest that, in a disadvantageous situation, argatroban was useful to maintain a sufficient total platelet count without a significant increase in bleeding risk.

## 6. Conclusions

Our study suggests argatroban could possibly be used, even without HIT occurrences, as a main strategy for systemic anticoagulation therapy during ECMO survival support; however, our preliminary data need to be confirmed in randomized controlled trials before widening the anticoagulant’s use.

## Figures and Tables

**Table 1:** Population characteristics. UFH: unfractionated heparin, ARG: argatroban, ECMO: extracorporeal membrane oxygenation, LOS-ICU: length of stay in the intensive care unit.

	UFH Group	ARG Group	*p*-Value
Number	40	21	
Age	55 (52–59)	56 (47–58)	0.750
Gender			0.870
Male	17	14	
Female	2	7	
ECMO days	10 (8–26)	31 (22–47)	0.001
Death	8	16	0.059
LOS-ICU	31 (18–39)	43 (32–56)	0.028
Anticoagulation days			
Heparin	13.58 (9.63–17.52)	15.28 (15.55–19.03)	0.520
Argatroban		17 (9.76–24.24)	

**Table 2:** Incidence of bleeding. The UFH column describes events under unfractionated heparin, and the argatroban column describes those that happened under argatroban.

Site of Hemorrhage	UFH	Argatroban	Odds Ratio	*p*-Value
Intracranial	3 (7.5%)	3 (14.2%)	0.52	0.405
Pulmonary (alveolitis)	6 (15%)	5 (23.8%)	0.63	0.488
Gastrointestinal	8 (20%)	4 (19.0%)	1.05	0.737
Site of cannulation	14 (35%)	4 (19.0%)	1.84	0.246
Tracheal stoma	14 (35%)	1 (4.8%)	7.29	0.011
High respiratory tract	7 (17.5%)	1 (4.8%)	3.65	0.243
Intermuscular	6 (15%)	3 (14.2%)	1.06	1.000
Total Bleeding Event/days of anticoagulant	58/579	21/357	1.73	0.041
Platelet transfusions per day of infusion	6/579	18/357	4.90	0.001
Fresh frozen plasma per day of infusion	16/579	18/357	1.82	0.082
Red blood cell unit per day of infusion	254/579	167/357	1.06	0.593

**Table 3:** Blood chemistry values sorted by group. aPTT is the activated partial thromboplastin time, PLT is the median platelet count during ECMO, PLT T0 is the platelet count on day 0, PLT T5 is the platelet count on day 5, and PLT drop is the difference in platelet count between day 5 and day 0. The statistical tests were conducted using the Wilcoxon rank-sum test. PLT = platelet, UFH = unfractionated heparin, ARG = argatroban, and PTT = activate prothrombin time.

Blood Test	UFH (Median + IR)	ARG (Median + IR)	*p*-Value
aPTT	42.65 s (37.25; 46.43)	44.70 s (36.70; 48.20)	0.443
PLT	138,500 cell/mm^3^ (100,000; 185,250)	80,000 cell/mm^3^ (52,500; 107,500)	0.001
PLT T0	271,000 cell/mm^3^ (188,500; 350,000)	59,000 cell/mm^3^ (42,000; 85,000)	0.001
PLT T5	177,500 cell/mm^3^ (115,500; 250,500)	67,000 cell/mm^3^ (46,000; 114,000)	0.001
PLT drop	−72,500 cell/mm^3^ (−135,500; −48,000)	7000 cell/mm^3^ (−3000; 16,000)	0.001
D-dimers	2935 ng/mL (2332; 4400)	6570 ng/mL (3170; 8590)	0.003

**Table 4:** Regression between PLT T5 and PLT T0 grouped for anticoagulants, where PLT T0 is platelet count on day 0, and anticoagulant is the treatment grouping. PLT T0 = platelet count at day 0, PLT T5 = platelet count at day 5.

PLT T5	Coefficient	Stand. Error	T	*p* > |t|	[95% Confidence Interval]
PLT T0	0.62	0.04	16.71	0.001	0.55, 0.69
Anticoagulant	30.50	16.99	1.80	0.078	−3.51, 64.50

R-squared: 0.83.

## Data Availability

Data are provided within the manuscript, and Supplementary Information files will be available upon explicit request to the corresponding author Alessia Mattei at her email address: a.mattei@policlinicocampus.it. All data are stored anonymously on a local server and on paper. A digital version of the data has been submitted as Supplemental Data.

## Supplementary Materials

The following supporting information can be downloaded at: https://www.mdpi.com/article/10.3390/jcm13226984/s1, Figure S1: Linear regression in Table 4: Residuals analysis.
