# Supplementary figures and images for: Anticoagulation Management for Veno-Venous ECMO in COVID-19 Patients: Argatroban as Rescue Therapy in Heparin-Associated Thrombocytopenia
Source: J Clin Med. 2024 Nov 20;13(22):6984. doi: 10.3390/jcm13226984 (PMC11595161; doi:10.3390/jcm13226984)

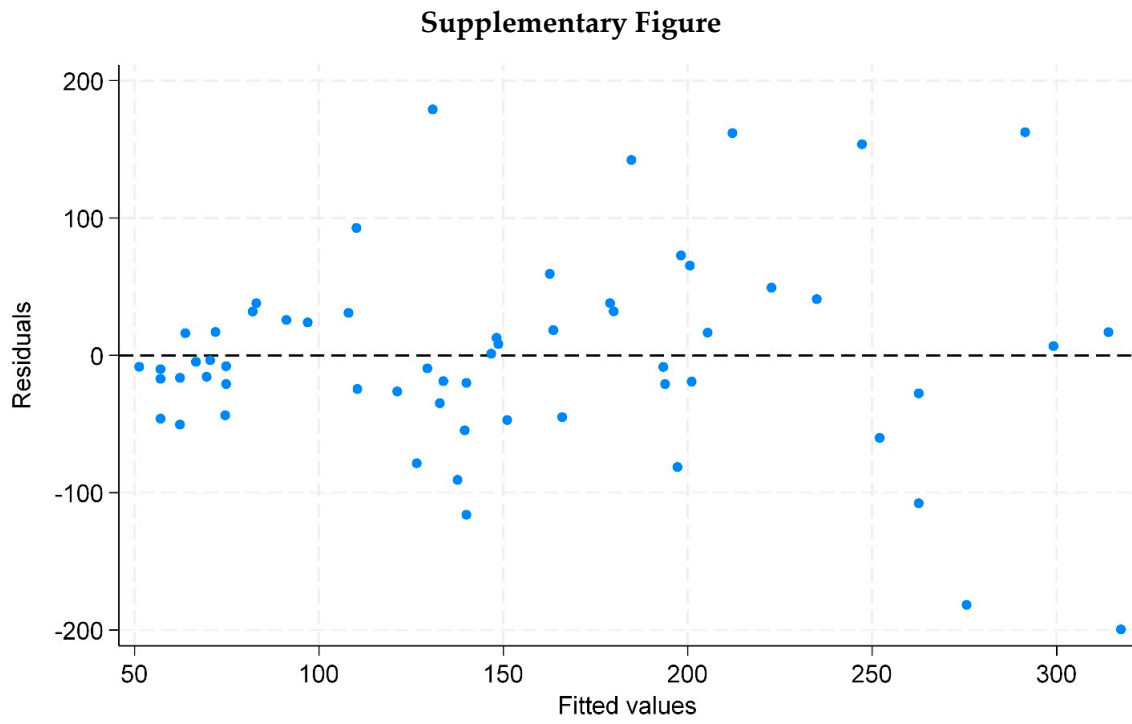

**Figure S1.** Linear regression in Table 4: residuals analysis.

Supplement: Supplementary file 1 [file jcm-13-06984-s001.zip › jcm-3291260-supplementary.pdf]
